# Factors affecting the polychlorinated biphenyl signatures in serum of adults living in a highly polluted area in eastern Slovakia

**DOI:** 10.3389/fpubh.2024.1477692

**Published:** 2024-10-30

**Authors:** Henrieta Hlisníková, Branislav Kolena, Tomáš Trnovec, Denisa Richterová Bagratuni, Henrieta Patayová, Kamil Čonka, Beata Drobná, Katarína Rausová, Juraj Tihányi, Soňa Wimmerová, Ida Petrovičová, Miroslava Nagyová, Ľubica Palkovičová Murínová

**Affiliations:** ^1^Department of Zoology and Anthropology, Faculty of Natural Sciences and Informatics, Constantine the Philosopher University in Nitra, Nitra, Slovakia; ^2^Department of Environmental Medicine, Faculty of Public Health, Slovak Medical University, Bratislava, Slovakia; ^3^Department of Toxic Organic Pollutants, Faculty of Medicine, Slovak Medical University, Bratislava, Slovakia; ^4^Institute of Health Protection, Faculty of Public Health, Slovak Medical University, Bratislava, Slovakia; ^5^Institute of Biophysics, Informatics and Biostatistics, Faculty of Public Health, Slovak Medical University, Bratislava, Slovakia

**Keywords:** polychlorinated biphenyls, congener, place of residence, food, age

## Abstract

**Introduction:**

Over the years eastern Slovakia has been subject to consistent monitoring of high levels of polychlorinated biphenyls (PCBs) in both the environment and human populations attributed to the former production of PCBs at the Chemko Strážske plant. We aimed to investigate the extent to which dietary habits and residential location could affect the concentrations of PCBs in the blood serum samples of subjects.

**Methods:**

We enrolled 602 adult subjects from eastern Slovakia with an average age of 45.14 (±8.49) years. To determine the concentrations of 21 PCB congeners, we used solid phase extraction along with gas chromatography coupled with high-resolution mass spectrometry. Based on questionnaire data, the assessment of dietary habits and residential location was performed using score calculations and creating a map.

**Results and discussion:**

Through principal component analysis, the 20 PCB congeners were classified into three groups: PC1, comprising highly chlorinated PCBs, and PC2 and PC3, consisting primarily of low chlorinated PCBs. Stepwise multivariate regression revealed positive and negative associations between PCB congeners represented by PC1-3 and scores related to the residential location and scores related to food consumption, respectively. We confirmed that levels of PCBs represented by PC1-3 increased with age. The geographical proximity to pollution sources proved to be a key contributing factor to the observed PCB levels in individuals residing in eastern Slovakia.

## 1 Introduction

Polychlorinated biphenyls (PCBs) are a class of chemical compounds in which 2–10 chlorine atoms are attached to the biphenyl molecule. There are 209 possible chlorinated compounds called congeners based on chlorine positions on the benzene ring (1, 2). During the 20th century, PCBs were extensively produced worldwide, primarily in the form of commercial mixtures like Aroclor^®^, Clophen^®^, Phenclor^®^, Kanechlor^®^, or Delor^®^. Consequently, exposure to PCBs typically occurs through mixtures of PCBs rather than individual congeners (3, 4). In addition, there is evidence that humans and animals are commonly exposed to mixtures of multiple persistent organic pollutants (POPs), such as PCBs, furans, and dioxins (5).

PCBs have been widely recognised as POPs due to their environmental persistence and adverse health effects (1, 2). PCBs are part of the Stockholm Convention on Persistent Organic Pollutants due to their toxicity. The treaty, adopted in 2001 and enforced in 2004, aims to eliminate or restrict the production and use of POPs, including PCBs, protecting human health and the environment (6).

PCBs are lipophilic, therefore, they can bioaccumulate in the food chain and the human body. Exposure to PCBs can result in adverse health effects (7). PCBs are categorized into two primary groups, dioxin-like (DL) and non-dioxin-like (NDL) PCBs, based on their chemical structure and different toxicological effects. DL PCBs possess a planar configuration, enabling them to bind to the aryl hydrocarbon receptor (AhR), similarly to compounds in the dioxin family. This initiates specific biological pathways related to the modulation of AhR signaling (8, 9). DL PCBs are generally regarded as more toxic, as evidenced by animal studies. They are involved in carcinogenesis, immunotoxicity, neurodevelopmental disorders, thyroid hormone disruption, reproductive toxicity, and hepatotoxicity. NDL PCBs exert their toxic effects via other mechanisms, such as oxidative stress, disruption of calcium signaling, and interaction with estrogen and androgen hormone receptors, as well as thyroid hormone transport proteins. Compared to DL PCBs, NDL PCBs have demonstrated more pronounced effects on neurodevelopment and thyroid hormone regulation, in addition to exhibiting reproductive toxicity and hepatotoxicity (9, 10).

Epidemiological studies from Taiwan showed that higher levels of PCBs were associated with more frequent medical problems, such as abnormal menstrual bleeding, skin diseases, goitre, anaemia, joint and spine diseases (11, 12). In addition, a study by Codru et al. (13) reported that there is a higher odd of being diagnosed with diabetes in adult Native Americans with higher levels of PCBs. The results of cohort studies from eastern Slovakia indicate the associations between exposure to PCBs and impaired cochlear function (14), neurodevelopment (15), immune (16), and thyroid function, and metabolic activity (17) in children and adults. Due to these concerns, their production has been banned or significantly restricted in many countries (1, 2).

Once released, PCBs strongly bind to soil and sediment, causing them to remain in the environment for extended periods, with most types breaking down over months to years. They slowly seep out of the soil, especially those more heavily chlorinated congeners, and their transfer to plants through soil is minimal. The process of PCBs moving through the environment includes their evaporation from land and water surfaces into the air, followed by their return to the earth through wet or dry falling, and then evaporation again. For the general population, inhalation of PCBs present in the air is one way people are exposed, besides ingesting PCBs through food (1). Animal-origin foods, such as milk, dairy products, eggs, and mussels, are particularly susceptible to contamination with persistent organic pollutants (POPs) due to their bioaccumulation potential. This has been demonstrated in the Province of Taranto, Southern Italy, where industrial activities have led to elevated levels of POPs, especially PCBs, in food products, with egg and mussel samples exceeding the EU’s recommended limits (18–21). Similarly, a study by Agudo et al. (22) examined serum PCB concentrations in adult participants from both northern and southern Spain as part of the EPIC-Spain cohort. They found that dietary habits, particularly fish consumption, were strongly associated with increased serum PCB levels, with notable regional differences in exposure. Further study using polytopic vector analysis (PVA) revealed distinct congener patterns across participants. The PVA identified five unique patterns of PCB mixtures, one of which resembled air samples collected near contaminated sediment deposits at Akwesasne, indicating possible recent inhalation exposure to volatilized Aroclor 1,248. Another pattern was linked to unaltered Aroclor 1,254, while a third resembled Aroclor 1,262 but lacked labile congeners. The remaining two patterns reflected subsets of major persistent congeners, suggesting differences in bioaccumulation profiles or individual toxicokinetics (23).

It has been shown that eastern Slovakia represented one of the most heavily polluted sites by PCBs worldwide (24–29). In high-volume ambient air samples collected in residential areas at 1 to 30 km from the former PCB plant Chemko (Michalovce District), extremely high PCB values of the sum of 6 PCBs concentrations of 1700 pg./m^3^ were found (26). Sediment samples from rivers and creeks collected in the Michalovce district showed contamination levels of 1,700–3,100 ng/g d.w. (total PCBs). Soils collected near the Chemko waste storage and dump site showed contamination levels of 400–5,800 ng/g (total PCBs) (24). Comparison of the mean serum concentration of the sum of PCBs of 2,871 ng/g lipids for adults (*n* = 149, age (mean ± SD) 45.3 ± 8.4) of Michalovce district in 2015 with the value of 3,105 ng/g lipids for a corresponding population sample (*n* = 1,008, age (mean ± SD) 44.6 ± 12.5) of Michalovce district in year 2001 (27) shows that the exposure of adults in Michalovce district decreased insignificantly over 14 years. An increasing emission trend of PCBs from 2010 to 2017 was observed (30). Despite the persistent source of environmental contamination, the storage of hundreds of tonnes of PCBs in the buildings of the former Chemko Strážske company had not been officially confirmed for years. In 2021, the removal of barrels with PCBs was initiated.

The exposure routes of the population living in PCB-polluted areas have not been comprehensively elucidated to date. Previous studies, such as Strémy et al. (28), did not find a significant association between food consumption and PCB serum concentrations in a 2016 analysis. Our current study expands on this by investigating additional and previously unaddressed factors contributing to PCB exposure, beyond dietary sources, for populations in eastern Slovakia near the Chemko plant. Using principal component analysis (PCA), we identified groups of PCB congeners based on their similarities and developed a novel scoring system to quantify exposure from multiple sources, including the frequency and origin of food consumption, as well as residential proximity to contamination sites. Additionally, we incorporated spatial analysis through mapping to assess the impact of geographical location on exposure risk. This approach not only provides deeper insights into localized PCB exposure pathways but also presents a scalable methodology that can be applied to other PCB-polluted regions globally. By identifying critical exposure factors, our findings offer valuable implications for understanding PCB contamination in various environmental contexts, contributing to broader efforts in mitigating PCB exposure worldwide. The findings have broader implications for understanding how environmental exposure to PCBs affects human health, especially in regions impacted by long-term industrial pollution.

## 2 Methods

### 2.1 Study population and sample collection, questionnaires

Within the project “PCB Exposure of Human Population Living in the Selected Regions of eastern Slovakia” (PCBExpo, funded by the Ministry of Health of the Slovak Republic, no. 2012/41-SZU-5), we recruited a total of 602 volunteers who had been permanent residents in one of the four districts around the area of Chemko plant: Michalovce, Vranov nad Topľou, Humenné, and Trebišov, for over 20 years. Subjects were approached and recruited for the study in the regional general practitioners’ outpatient clinics. Each participant was presented with detailed information about the project and its aims. The number of subjects was evenly distributed, with 150 individuals from each district. Exclusion criteria for participation in the study have been described previously (28).

Each participant underwent a blood sampling procedure. Details regarding the handling of specimens and isolation of serum can be found in the study by Jusko et al. (31). The research was conducted with the approval of the Institutional Review Board (IRB) at the Slovak Medical University (Approval from November 18, 2013). IRB reviewed the project design, informed consent, information for participants and general questionnaire. Participation in the study was anonymous and voluntary, and all subjects provided informed consent before their involvement.

Upon enrolment, the subjects filled in a general questionnaire regarding sociodemographic data and a modified version of the food frequency questionnaire (consumption of food with potentially higher levels of PCBs such as consumption of poultry, pork, beef, eggs, lard, milk and dairy products and also the origin of food) by Sonneborn et al. (59) administered by skilled personnel.

### 2.2 Calculating of score of exposure to PCB

To calculate a score of PCB exposure, we utilized data obtained from the questionnaire, which included information on the frequency of food product consumption, the origin of food products, and the residency location. The scoring system of the frequency of food product consumption was as follows: never (0 points), monthly (0.33 points), weekly (0.66 points), and daily (1.00 point). We considered a higher frequency of consumption of poultry, pork, beef, eggs, dairy products, or milk to indicate a higher risk of PCB exposure. By summing the scores of all items, we calculated a score for the frequency of food product consumption.

Similarly, we assigned scores for the origin of food products. The scoring system for this category was as follows: retail food products (0.33 points), a combination of retail and homemade food products (0.66 points), and homemade products (1.00 point). We considered the consumption of homemade products to pose a higher risk of PCB exposure, as they were grown in an area with higher environmental concentrations of PCBs compared to other regions of Slovakia (32). Again, the scores of all items were summed to calculate the score for the origin of food products.

**Figure 1:**
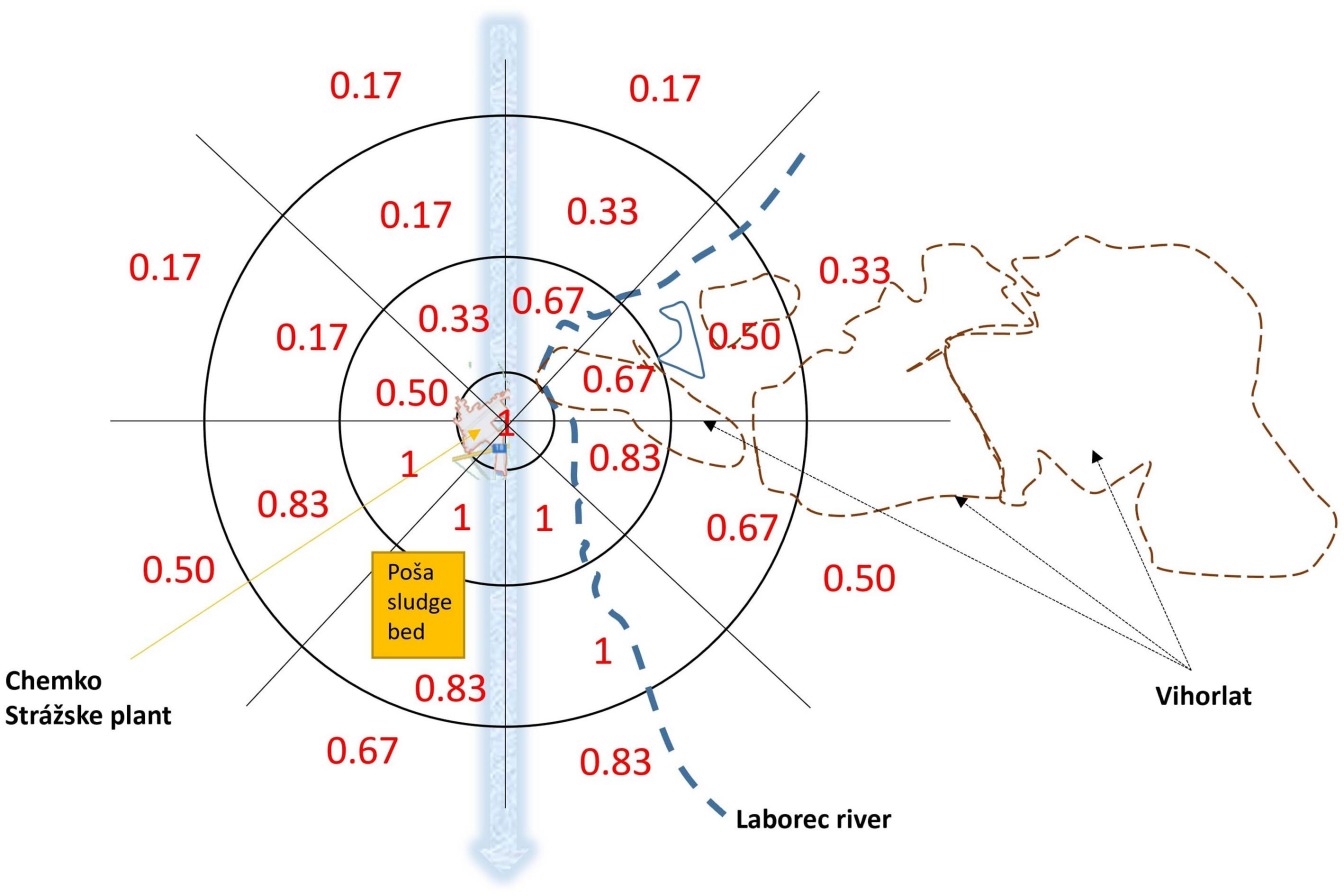
Score that expresses the potential level of exposure to the distance from Chemko Strážske plant, Poša sludge bed, the direction of wind flow, the stream of Laborec river and the georelief of Vihorlat mountains.

We then evaluated the geographical proximity in relation to the Chemko Strážske plant, which is a potential source of PCB exposure. Additionally, we considered the location of the Poša sludge bed for Chemko Strážske, as well as the weather and geomorphological characteristics of the area, including wind direction (north–south direction), the stream direction of the Laborec river, and the Vihorlat mountain range. These factors could potentially influence the exposure to PCBs in our study subjects. The score for location of residence ranged from 0.17 (the lowest score) to 1.00 point (the highest score). Figure 1 depicts a schematic illustration of the cartographic display and scoring methodology. The innermost circle on the map represents a radius of 0–2 km around the Chemko Strážske plant, followed by the radius of 2.1–7.4 km, 7.5–13.5 km, and 13.6–22.5 km. The outermost circle represents a radius of more than 22.6 km around the Chemko Strážske plant. The map scale was 1:500,000.

### 2.3 PCB and lipid measurement, quality assurance and quality control

Concentrations of 21 specific PCB congeners (IUPAC numbers 28, 52, 74, 99, 101, 105, 114, 118, 123, 138, 153, 156, 157, 167, 170, 180, 187^+182^, 189, 194, 196^+203^, and 199^+201^) were determined in blood serum samples using solid phase extraction and high-resolution mass spectrometry coupled to gas chromatography, following the methods described in a previous publication (33). The mass spectrometry measurements were performed by isotope dilution method using 13C labeled extraction standard solutions on a HP 6890 GC (Hewlett–Packard, Palo Alto, CA, United States) coupled to a Finnigan MAT95XP (Thermo Fisher Scientific, Bremen, Germany) and on a Trace GC Ultra coupled to a Thermo DFS (Thermo Fisher Scientific, Bremen, Germany). GC separation was carried out on a DB-5MS column (60 m, 0.25 mm i.d., 0.25 μm film thickness, J&W Scientific, Folsom, United States). Recovery standards of 13C labeled PCBs were added to the fractions prior to a mass spectral analysis. An analysis batch consisted of 10 serum samples and a solvent blank. Certified human serum samples (SRM 1589a, NIST, Gaithersburg, MD, United States) were analysed to check the analytical method quality. The analytical method was completely verified (including recoveries, accuracy, extended measurement uncertainty, etc.) using more than 10-times repeated analysis of a certified reference material (SRM Organics in Cod Liver Oil 1588b, NIST, Gaithersburg, MD, United States). Control charts were plotted for QC samples, blanks, and verification calibration standards as a basis for continuous checking of accuracy, precision, and reliability of the analytical process.

All analytical measurements were performed at the National Reference Centre for Dioxins and Related Compounds, which is part of the Department of Toxic Organic Pollutants at the Slovak Medical University. The laboratory is certified by the Slovak National Accreditation Service (ISO/IEC 17025:2017, certification no. S-111) and regularly participates in interlaboratory studies and proficiency tests for dioxins and PCBs in food and feed organized by the EU-RL for Halogenated Persistent Organic Pollutants in Feed and Food (Freiburg, Germany) and the interlaboratory comparison program for PCBs and OCPs in blood serum (G-EQUAS) organized by the Institute and Out-Patient Clinic for Occupational, Social, and Environmental Medicine of the Friedrich-Alexander University in Erlangen, Germany. The accredited laboratory also routinely participates in proficiency tests designed for environmental matrices (sediment) such as InterCinD organized by Labservice Analytica s.r.l. (Italy), WEPAL / QUASIMEME organized by Wageningen University & Research (Netherlands) and others.

To account for individual variations in serum lipid levels, the total serum lipids were estimated using the enzymatic summation method, as described by Akins et al. (34). The reported PCB concentrations in this study have been adjusted for serum lipid content.

### 2.4 Data analysis

Prior to the statistical analysis, concentrations of PCB congeners were log-transformed due to the non-normal distribution of the data. For PCB congeners with values below the limit of detection (LOD), two approaches were used depending on the proportion of values below the LOD. If fewer than 20% of values were below the LOD, the LOD value was divided by the square root of two. For congeners with more than 20% of values below the LOD, the LOD value was divided by two. This approach is based on previous studies by Persky et al. (60) and Weisskopf et al. (61). Missing data were handled in the IBM SPSS Statistics by assigning a discrete value to represent them.

Pearson correlation analysis, unpaired *t*-test, and one-way analysis of variance (ANOVA) were conducted to identify potential confounding variables based on the baseline characteristics of the subjects. Variables that were found to be statistically significant (*p* ≤ 0.05) potential confounding variables included sex, age, active and second-hand smoking, alcohol consumption, work in the industry, and contact with toxic material.

Principal Component Analysis (PCA) with direct oblimin rotation was employed to reduce the number of correlated polychlorinated biphenyl (PCB) congeners (*n* = 21) and identify patterns in the data by addressing correlations between the 21 measured congeners. The PCA approach reduces the dimensionality of the dataset while preserving as much variability as possible by identifying new, uncorrelated variables, called principal components (PCs), which explain the maximum variance in the data (35). Eigenvalues greater than one were used as the criterion for selecting principal components to select components that explain a significant portion of the variance. Direct oblimin rotation was utilized to simplify and clarify the data structure (36). The analysis identified three PCs, which explained 85.89% of the total variance in PCB congener concentrations.

Based on the PCA results, molar sums of the PCB congeners corresponding to these three PCs were calculated. These molar sums were then used as independent variables in a stepwise multivariate regression analysis.

Stepwise multivariate regression analysis combined with Akaike information criterion (AIC) (37) was employed to examine associations between the molar sums of PCB congeners and the scores for residency location, as well as the consumption of food products and all potential confounding variables identified in previous analyses.

All statistical analyses were performed using IBM SPSS Statistics (version 21.0; SPSS Inc., Chicago, IL, United States) for PCA and for potential confounder identification. The R package “MASS” was used to conduct stepwise multivariate regression with AIC (37). A *p*-value of ≤0.05 was considered statistically significant.

## 3 Results

**Figure 2:**
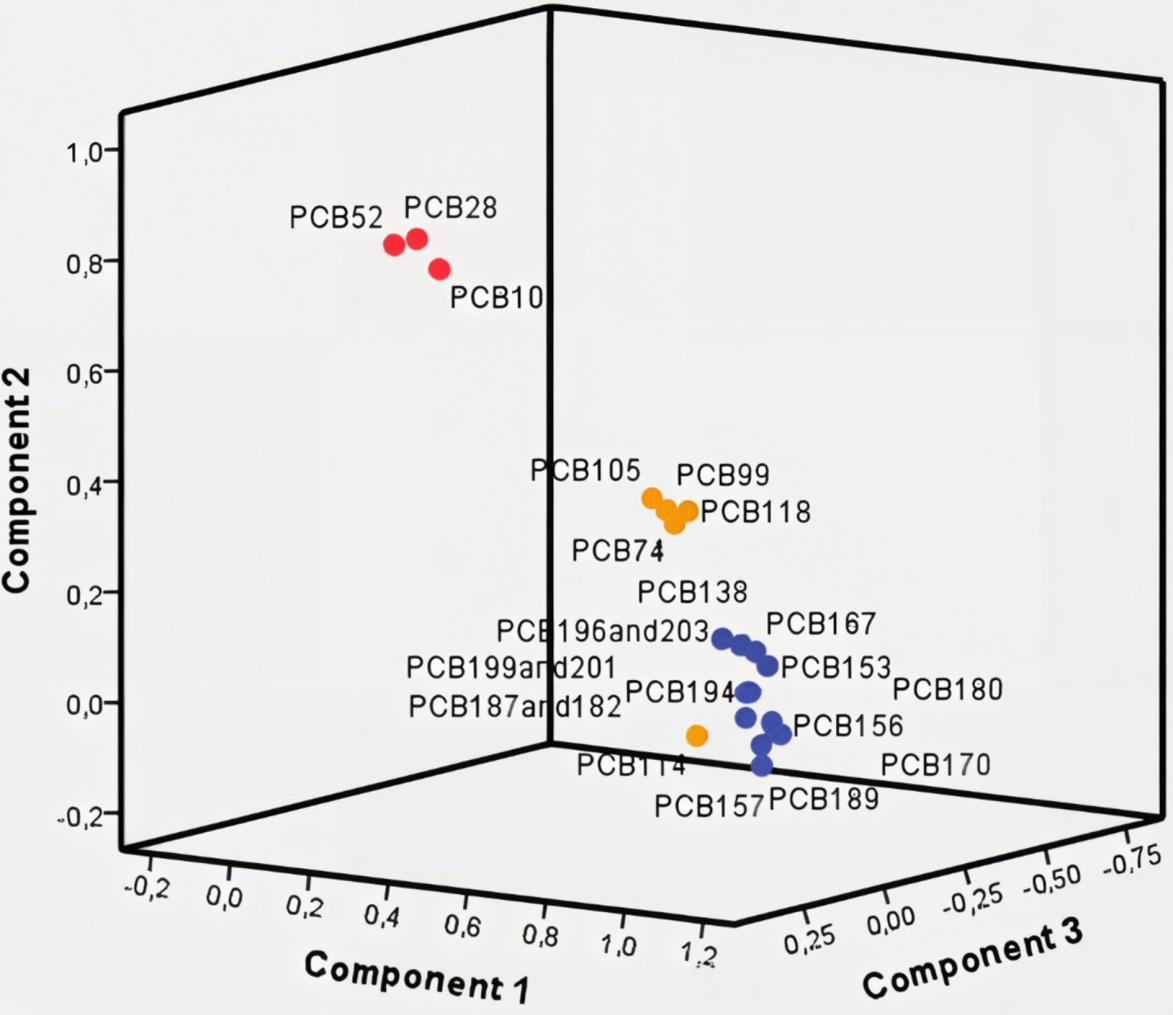
Component plot of PCB congeners: blue bullets represent PCB congeners of PC1, red bullets are PCB congeners of PC2, and yellow bullets are PCB congeners of PC3.

Baseline characteristics of subjects are shown in Table 1. Figure 2 presents the component plot of 20 PCB congeners analysed in this study. PCB 123 did not show a significant contribution to any of the three principal components in this PCA analysis. The analysis resulted in three principal components (PCs), which accounted for 72.03% (PC1), 8.72% (PC2), and 5.14% (PC3) of the total variance. PC1 was found to be positively controlled by PCB congeners with six or more chlorine atoms, including PCB 138, PCB 153, PCB 156, PCB 157, PCB 167, PCB 170, PCB 180, PCB 187^+182^, PCB 189, PCB 194, PCB 196^+203^, and PCB 199^+201^. These congeners are represented by blue bullets in the component plot. PC2 was positively controlled by PCB congeners with three to five chlorine atoms, specifically PCB 28, PCB 52, and PCB 101, which are indicated by red bullets in the plot. PC3 was positively controlled by PCB congeners with four to five chlorine atoms, including PCB 74, PCB 99, PCB 105, PCB 114, and PCB 118. These congeners are represented by yellow bullets in the plot.

**Table 1:** Baseline characteristics of the subjects (*n* = 602).

		Mean (±SD)	% (*n*)
Sex	Female		50.80 (306)
	Male		49.20 (296)
Age (years)		45.14 (±8.49)	
BMI (kg/m^2^)		27.52 (±4.61)	
Active smoking	Yes		27.25 (164)
	No		19.10 (115)
	Missing		53.65 (323)
Passive smoking	Yes		21.76 (131)
	No		77.08 (464)
	Missing		1.16 (7)
Alcohol consumption	Yes		67.44 (406)
	No		30.23 (182)
	Missing		2.33 (14)
Education	Elementary and lower secondary education		35.55 (214)
	Higher secondary education		44.68 (269)
	College/University		19.44 (117)
	Missing		0.33 (2)
Work in the industry	Yes		78.20 (471)
	No		21.80 (131)
	Missing		0.00 (0)
Contact with hazardous materials	Yes		83.40 (502)
	No		16.60 (100)
	Missing		0.00 (0)
Score of frequency of food products consumption		5.94 (± 0.74)	
	Missing		1.83 (11)
Score of origin of food products		8.45 (± 1.37)	
	Missing		1.83 (11)
Score of location of residence		0.45 (± 0.25)	
	Missing		1.83 (11)
Arbitrary score of exposure		14.84 (± 1.64)	
	Missing		1.83 (11)

**Figure 3:**
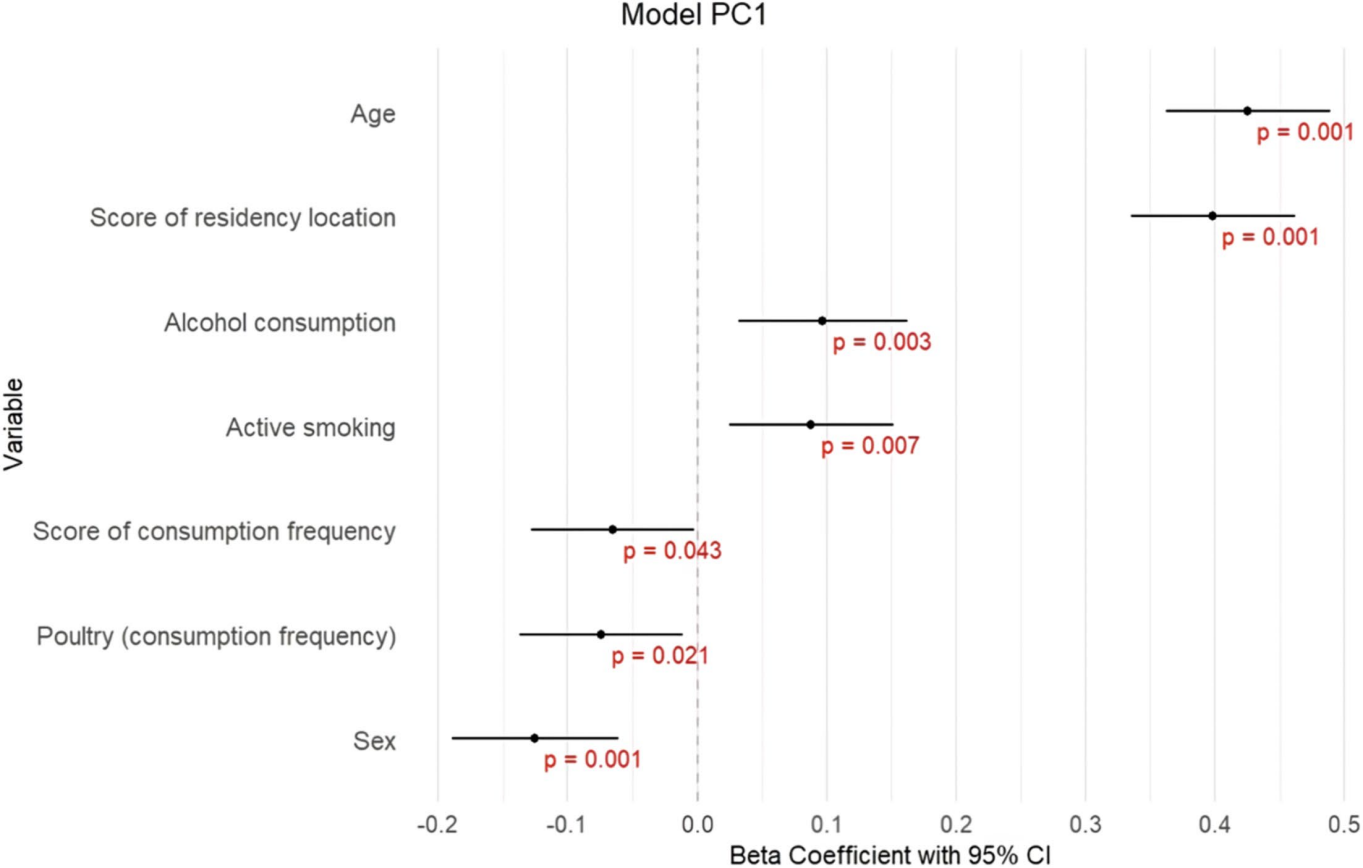
Graphical visualization of stepwise linear regression for model PC1.

**Figure 4:**
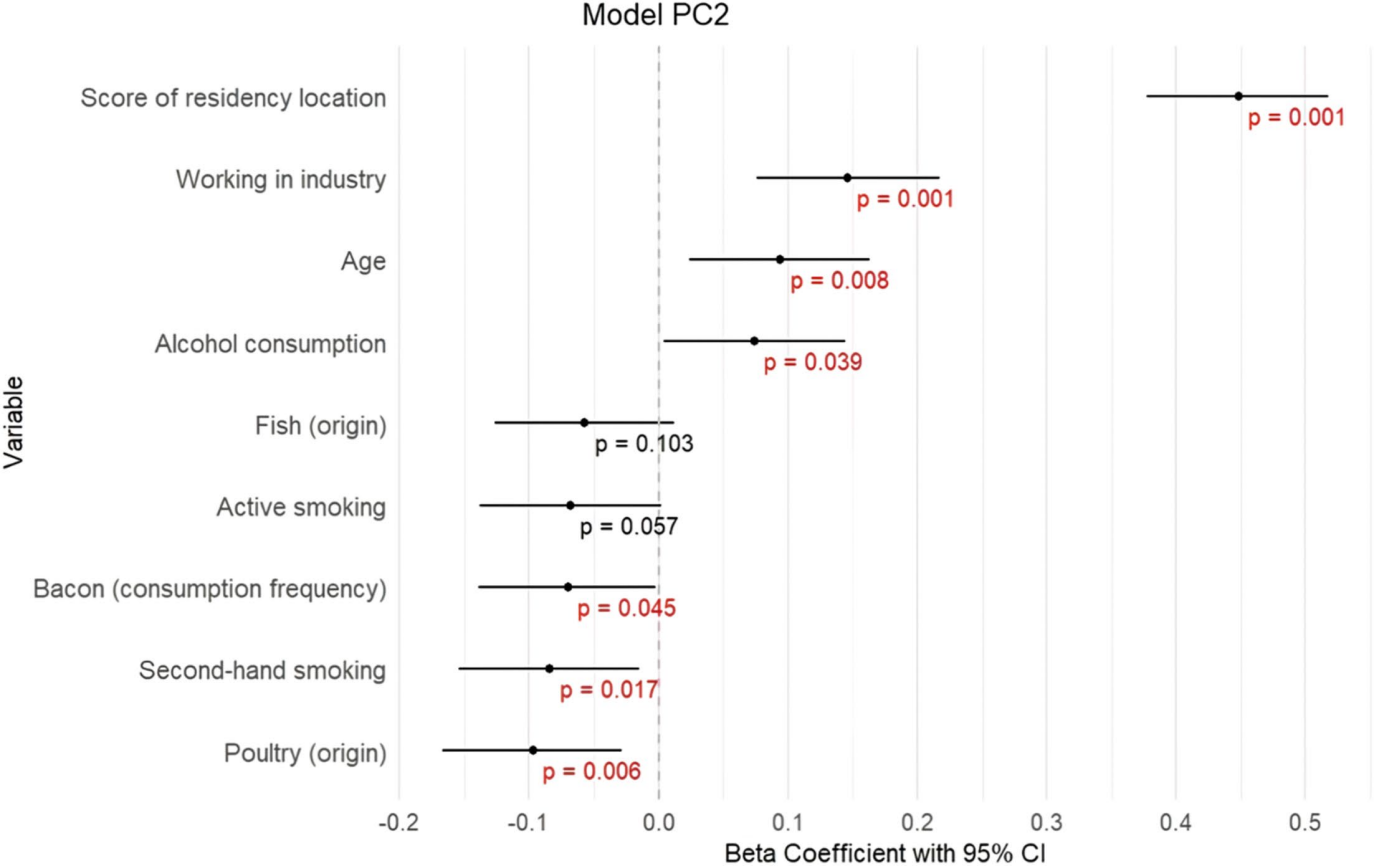
Graphical visualization of stepwise linear regression for model PC2.

**Figure 5:**
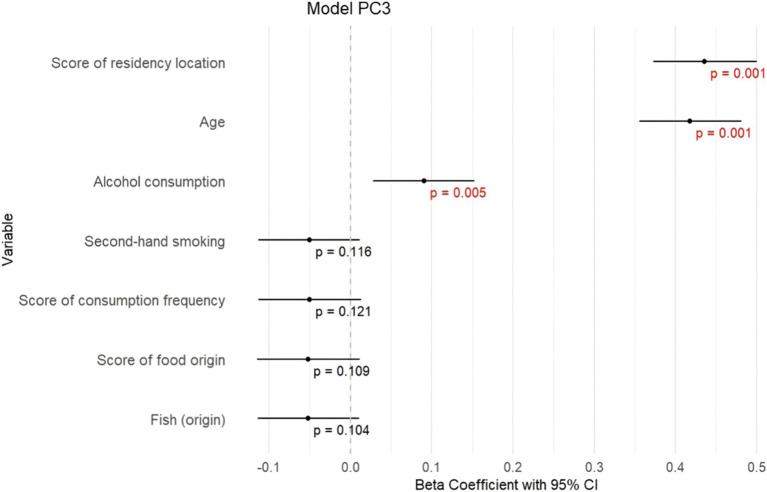
Graphical visualization of stepwise linear regression for model PC3.

To identify the key factors contributing to PCB exposure, a stepwise multivariate regression analysis was conducted, examining the associations between the sums of PCB congeners represented by PCs and diet, residency location, and potential confounding variables – sex, age, active and second-hand smoking, alcohol consumption, work in the industry, and contact with toxic material. The following models were chosen according to the lowest AIC values as the models with the best fit:

1. In the first model (Figure 3; Supplementary Table S1), ΣPC1 showed significant positive associations with the score of residency location (*β =* 0.398, *p ≤* 0.001), age (*β =* 0.425, *p ≤* 0.001), alcohol consumption (*β =* 0.097, *p* = 0.003), active smoking (*β =* 0.088, *p* = 0.007); and negative associations with sex (*β =* −0.126, *p ≤* 0.001), poultry consumption (*β =* −0.074, *p* = 0.021) and score of consumption frequency (*β =* −0.065, *p* = 0.043). These results indicate that higher levels of PCB congeners represented by ΣPC1 are observed in subjects living in proximity to Chemko plant, in subjects with higher age, higher alcohol consumption, in smokers and males, and in subjects with lower consumption frequency of food, especially poultry.
2. In the second model (Figure 4; Supplementary Table S2), ΣPC2 exhibited significant positive associations with the score of residency location (*β =* 0.135, *p ≤* 0.001), age (*β =* 0.094, *p* = 0.008), working in industry (*β =* 0.146, *p ≤* 0.001), alcohol consumption (*β =* 0.074, *p* = 0.039) and negative associations with second-hand smoking (*β =* −0.084, *p* = 0.017), bacon consumption (*β = −*0.070, *p* = 0.045) and poultry consumption (*β =* −0.097, *p* = 0.006). Fish consumption and active smoking are also included in this model, however, with an insignificant contribution. This indicates that higher levels of PCB congeners represented by ΣPC2 are observed in subjects living in proximity to the Chemko plant, in subjects with higher age, alcohol consumption, and in workers in industry. Lower levels of PCB congeners represented by ΣPC2 are observed in second-hand smokers and subjects that consumed poultry from retail and higher consumption of bacon.
3. In the third model (Figure 5; Supplementary Table S3), ΣPC3 showed significant positive associations with age (*β =* 0.418, *p ≤* 0.001), alcohol consumption (*β =* 0.091, *p =* 0.01) and the score of residency location (*β =* 0.436, *p ≤* 0.001). Fish consumption, score of consumption frequency, score of food origin and second-hand smoking are also included in this model, however, with insignificant contribution. These results indicate that higher levels of PCB congeners represented by ΣPC3 are observed in older subjects, with higher alcohol consumption and living in proximity to the Chemko plant.

**Figure 6:**
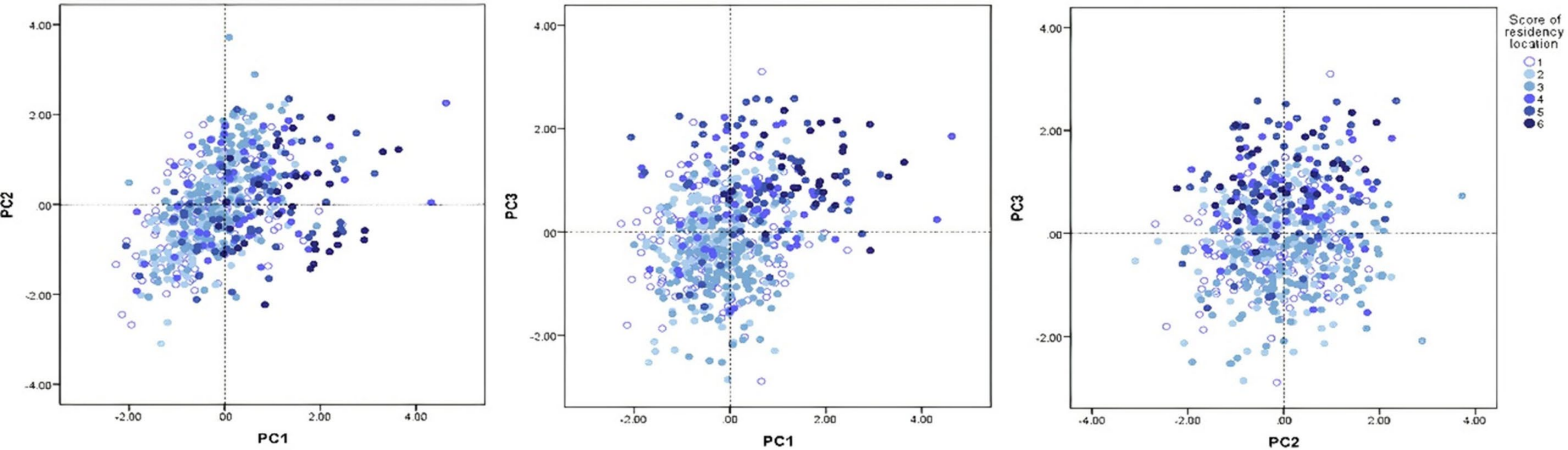
Score plots for PCs according to the score of residence location, where a score of 1 (represented by white) indicates the lowest value, and a score of 6 (represented by dark blue) indicates the highest value..

**Figure 7:**
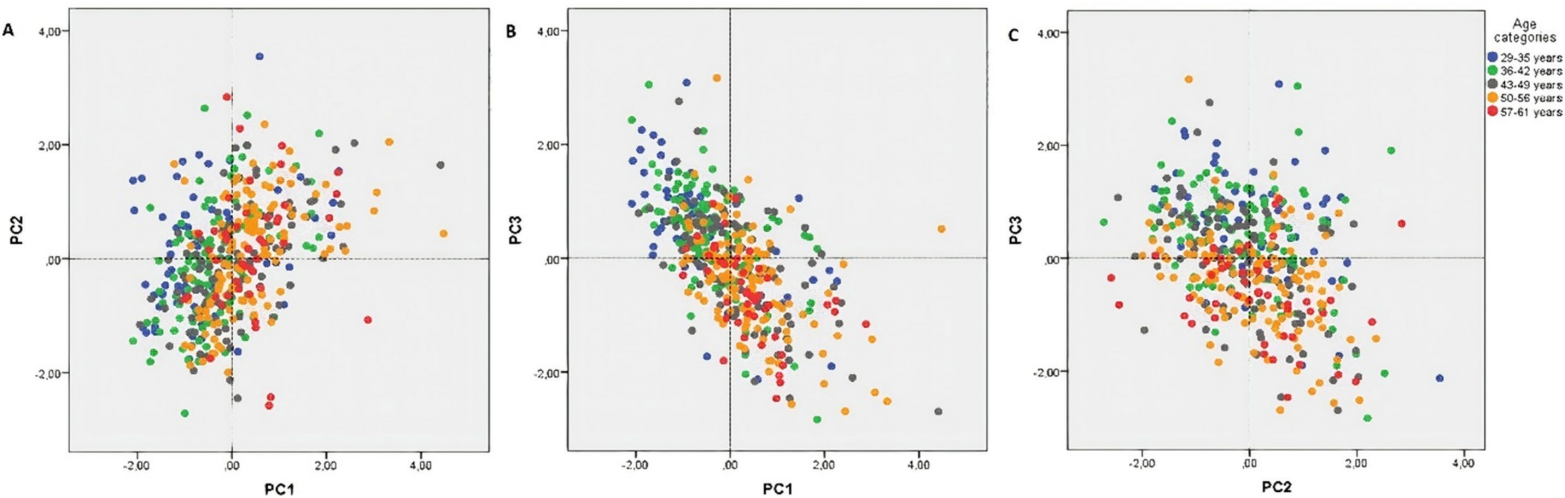
Score plots for PCs according to age categories: blue: 29–35 years, green: 36–42 years, gray: 43–49 years, orange: 50–56 years, red: 57–61 years.

As the results of stepwise multivariate regression showed, the score of residency location and age of our subjects are the two strongest predictors of PCB concentrations represented by PC1-3. Figure 6 illustrates the scores plots showing the mild incline of the score of residency location along the PC1 and PC3 axes. Based on these scores plots, it can be inferred that subjects with higher scores for residency location were predominantly exposed to congeners from the PC1 and PC3 groups. Conversely, subjects with lower scores for residency location were mainly exposed to PCB congeners from the PC2 group. This distinction in exposure patterns underscores the importance of residency location in the distribution of PCB congener profiles. Next, the PCA score plots for PC1 and PC2, as well as PC2 and PC3 (Figures 7A,C), did not reveal any clear patterns regarding the association between age and these PCs. However, the PCA score plot for PC1 and PC3 (Figure 7B) showed a noticeable pattern. It indicated that older individuals tended to have higher exposure to PCB congeners represented by PC1, while younger individuals had relatively higher exposure to PCB congeners represented by PC3.

## 4 Discussion

To explore the potential relationship among specific PCB congeners in our study, we conducted PCA. The PCA yielded three PCs, with PC1 influenced by highly chlorinated PCBs, while low chlorinated PCBs influenced PC2 and PC3. Previous studies utilising PCA to identify PCB components have also observed that the categorisation of PCBs is mainly based on their physicochemical properties, precisely the number and location of chlorine atoms. In a study by Ravenscroft et al. (38) at the Akwesasne Mohawk Nation, United States, PCA analysis resulted in four components. PC1 consisted of PCB congeners with lower levels of chlorination (52, 70, 90_101, and 123_149), explaining 50.1% of the variance. PC2 encompassed highly chlorinated congeners (164_138, 153, 180, 99, and 187), explaining 15.9% of the variance. Additionally, PC3 and PC4 included highly chlorinated congeners (105, 118, and 110, as well as 153, 164_138, and 99), each contributing to 6% of the variance. Another study investigating PCB exposure in a heavily contaminated area in Anniston, United States, observed a similar pattern. The results of the PCA analysis revealed the division of PCB congeners into four groups based on their degree of chlorination. Group A consisted of congeners 28, 66, and 74; group B consisted of congeners 99, 118, 138, 146, 153, 167, 183, and 187; and groups C1 and C2 comprised highly chlorinated congeners (157, 156, 170, 177, 178, 180, 189, 194, 196, 199, 206, and 209). Furthermore, an observation indicated that older individuals manifested a greater incidence of highly chlorinated PCBs (39). Similarly, Megson et al. (40) employed PCA to examine the distribution of PCB congeners based on their degree of chlorination. Group 1 consisted of low-chlorinated congeners (28, 44, 49, 52, 66, 87, 101, 110, 149, and 151), predominantly found in younger subjects. Group 2 comprised highly chlorinated congeners (156, 170, 172, 180, 194, 195, 199, 203, 206, and 209). In a study by Plaku-Alakbarova et al. (41), the PCA results demonstrated the division of PCB congeners into various groups based on the number and position of chlorine atoms. Groups 4, 7, and 1 contained tri/tetra-, tetra/penta-, and hexa/hepta-chlorinated PCBs with chlorine atoms at the 4,4′ positions. Groups 2, 8, and 5 contained tetra- to hexa-, hexa/hepta-, and hepta/octa-chlorinated PCBs with chlorine atoms at the 2,2′ positions.

Several regions in the world have experienced significant PCB contamination due to industrial activities, such as Anniston, Alabama, United States (42); Grimsby, Ontario, Canada (43); Lanzhou, China (44); or Thane Creek, India (45). The next notable source of PCB exposure was the Chemko Strážske plant in eastern Slovakia. The plant produced PCBs utilized as insulating liquids and plasticisers in paints, adhesives, and seals. Between 1959 and 1984, the total production of PCBs at the Chemko Strážske plant reached 21,500 tons, and a significant amount of hazardous waste from this production is still being stored. Furthermore, a substantial quantity of PCBs was released into the environment during manufacturing, leading to extensive contamination of water, soil and living organisms in eastern Slovakia (26, 32). To investigate the relationship between PCB components and residency location, we calculated a residence score for the study subjects based on their residence. We devised an original approach (Figure 1), allowing us to assess the exposure to PCBs related to the locality of residence not only according to the proximity to Chemko Strážske but also by considering other factors such as the distance from Poša sludge bed, the direction of wind flow, the stream of Laborec river and the relief of Vihorlat mountains, that can significantly modulate the level of exposure. In such a way, we could more accurately evaluate the impact of these multifaceted elements on PCB exposure. The results demonstrated a positive association between the PCB components and the residence score, indicating that individuals residing in closer proximity to Chemko Strážske (considering also environmental factors mentioned above) showed higher levels of PCBs. In addition, the score plots (Figure 6) showed that subjects with higher scores for residence location were predominantly exposed to PCB congeners from the PC1 and PC3 groups. Conversely, subjects with lower scores for residence location were exposed to PCB congeners from the PC2 group. We assume that subjects living near the Chemko plant had higher levels of PCBs with highly chlorinated congeners from PC1, affected by natural sources, such as river flow, the prevailing direction of wind currents, and the mountainous terrain. Subjects living far from the Chemko plant had higher levels of PCBs with low chlorinated congeners from PC2. The volatility of low chlorinated congeners of PCBs can explain this. Therefore, they can reach a greater distance (28, 46). Previous research has indicated that areas near the Chemko Strážske plant in eastern Slovakia exhibit higher levels of pollution compared to more distant regions (28, 29). Years ago, a team of researchers from Slovakia analysed air, water, soil, and sediment samples in the area of Chemko Strážske. The results indicated substantially heightened PCB levels in the vicinity of the Chemko Strážske region, especially near the Laborec river, which receives effluent discharge from the plant, as well as within the Zemplínska Šírava reservoir. Research also pointed out that these elevated concentrations impacted aquatic life and wildlife in rivers and forests (26, 47).

Prior investigations in eastern Slovakia have unveiled that domestically produced food items, particularly meat and eggs, exhibit elevated levels of PCB contamination when juxtaposed with commercially available products (32). Studies from highly contaminated areas with PCBs, such as Anniston in Alabama (USA) and Lanzhou (China), confirmed the findings from Slovakia that contaminated food, mainly fish, meat, and eggs, can be a source of PCB exposure for residents living near industrial areas (42, 44). Building upon this knowledge and previous research findings, we evaluated our subjects’ consumption behavior to the concentrations of PCB congeners. We also assigned each study participant a food origin score and consumption frequency score based on the questionnaire. Those who consumed homemade food products and/or consumed with higher frequency received a higher score, while those consuming retail products and/or consumed with lower frequency received a lower score. The hypothesis stating that homemade food products and high food consumption frequency are associated with higher levels of PCBs in our subjects was not confirmed by our results. On the contrary, we did observe statistically significant negative associations between the ΣPC1, ΣPC2 and the consumption frequency score or food origin score among the subjects. Our results are not in line with the previous research on the population from eastern Slovakia (28); there was no significant association between levels of PCBs in human blood serum and the origin of food. On the contrary, studies by Donato et al. (48) and Helmfrid et al. (49) reported a positive association between PCB exposure and food consumption frequency of probands from highly polluted areas in Italy and Sweden, respectively.

Based on the findings obtained through stepwise multivariate regression analysis, the place of residence could impact PCB exposure more than food origin or food consumption frequency in subjects residing in eastern Slovakia. This suggests that inhalation of contaminated air might be a potential route of PCB exposure, as was shown in previous studies (28, 29, 43). While PCBs are commonly recognised for their tendency to bioaccumulate in the food chain, they can also enter the air through various industrial processes, combustion, and volatilisation from contaminated sources. Once released into the atmosphere, PCBs can be transported over long distances and eventually settle on surfaces such as soil and water. Exposure to airborne PCBs can occur in different scenarios. Individuals residing near industrial facilities involved in the production, usage, or disposal of PCBs may experience heightened levels of PCB-contaminated air. Furthermore, regions with a history of PCB contamination, such as hazardous waste sites or heavily industrialized areas, may exhibit elevated concentrations of airborne PCBs (28, 29, 50, 51).

Our findings showed that age is a significant factor in PCB levels in the serum of subjects. Our study aligns with previous research by Jursa et al. (25), Megson et al. (40), and Pavuk et al. (42) confirming a positive association between PCB levels and the age of the study subjects. This association can be attributed to two main factors: bioaccumulation of PCBs in the human body and slower metabolic activity in older individuals (52, 53). The historical use and accumulation of PCBs in the environment is a primary reason for higher PCB levels in older individuals. PCBs were extensively used in various industrial applications until their restrictions or bans. This led to the widespread release of PCBs into the environment through industrial processes and waste disposal. As a result, older generations had greater opportunities for exposure to PCBs over a more extended period. PCBs resist environmental degradation and persist in the environment, allowing them to bioaccumulate in organisms, including humans, through the food chain (1, 2). The second factor contributing to higher PCB levels in older individuals is slower metabolic activity. Research indicates that older humans metabolize PCBs more slowly than younger individuals (53–56). Metabolism of PCBs involves enzymatic processes, including oxidation and conjugation, which facilitate their breakdown and elimination from the body. Age-related changes in liver function, such as reduced enzyme activity, can contribute to a slower metabolism of PCBs in older individuals. Certain enzymes involved in PCB metabolism, such as cytochrome P450 enzymes, may exhibit decreased activity with age. These enzymes are crucial in transforming PCBs into more easily excreted metabolites (52, 53). The slower metabolism of PCBs in older individuals results in prolonged exposure to these compounds and an increased risk of accumulation in the body. In summary, the higher levels of PCBs in older individuals can be attributed to the historical accumulation of PCBs in the environment and the slower metabolic activity associated with aging. This interplay of factors fosters the enduring presence and accumulation of PCBs within the bodies of older people (53–56). Moreover, score plots for PCs according to age groups (Figure 7B) showed that older subjects had higher concentrations of highly chlorinated congeners of PCBs compared to the younger subjects. We confirmed the findings of Megson et al. (40) and Pavuk et al. (39) which can be explained by the longer persistence of highly chlorinated congeners of PCBs in the human body (46, 57).

The strengths of our study lie in the clear PCB exposure scoring methodology, which incorporates a comprehensive scoring system for food origin, frequency of consumption, and residential location of subjects. The development of a map that accounts for multiple environmental and geographic factors in determining the residential score represents an innovative approach as well.

It is important to note that our study has some limitations related to the recruitment strategy, potentially impacting the generalizability of the study results. Selection bias might have occurred by recruiting subjects solely from outpatient clinics. These individuals could differ systematically from the general population. Recall bias might have occurred when filling out the questionnaire, and subjects did not remember their consumption behavior in detail. Non-response bias may be present, as subjects who chose not to participate in our study could differ systematically from those who did (58). These factors could limit the generalizability of the study results.

## 5 Conclusion

Our study provides insights into the origins, pathways, and determinants of PCB exposure, with a focus on the population of eastern Slovakia, though its implications are also applicable for other highly polluted regions in the world. By analysing PCB congeners in detail, we identified the significant role of residence proximity to contamination sources, age, and lifestyle factors like smoking and alcohol consumption in influencing PCB levels. Our novel mapping approach, which considers environmental factors such as wind direction, river flow, and mountainous terrain, offers a more precise framework for assessing exposure, applicable to other regions worldwide facing PCB contamination.

The findings underscore the global relevance of residency location in determining PCB exposure levels, as individuals near industrial contamination sites, such as the Chemko Strážske plant, are particularly vulnerable to higher concentrations of highly chlorinated PCBs. This pattern is not unique to Slovakia and mirrors trends observed in other heavily contaminated regions around the world, including the USA, China, and other industrialized areas. Furthermore, our study suggests that inhalation of contaminated air could be a critical exposure pathway for PCB congeners in many parts of the world.

Given the persistence of PCBs in the environment and their ability to bioaccumulate, these results highlight the need for global, knowledge-based management strategies to address historical contamination, mitigate exposure, and protect vulnerable populations. The data generated in this study not only enhance our understanding of PCB distribution in a local context but also serve as a foundation for broader public health interventions aimed at reducing PCB exposure and its associated health risks worldwide.

## Data Availability

The raw data supporting the conclusions of this article will be made available by the authors, without undue reservation.
