# Supplementary material for: Factors affecting the polychlorinated biphenyl signatures in serum of adults living in a highly polluted area in eastern Slovakia
Source: Front Public Health. 2024 Oct 30;12:1477692. doi: 10.3389/fpubh.2024.1477692 (PMC11558523; doi:10.3389/fpubh.2024.1477692)
Supplement: Supplementary file 1 [file Data_Sheet_1.docx]

**Table S1** Stepwise regression model of predicting variables for molar sum of PCB congeners represented by PC1.

| **Model: ΣPC1** | ***β*** | **95% CI** | | ***p*** |  |
| --- | --- | --- | --- | --- | --- |
| **Sex** | **-0.126** | **-0.190** | **-0.061** | **≤0.001** | *** |
| **Age** | **0.425** | **0.362** | **0.489** | **≤0.001** | *** |
| **Active smoking** | **0.088** | **0.025** | **0.152** | **0.007** | ** |
| **Alcohol consumption** | **0.097** | **0.032** | **0.162** | **0.003** | ** |
| **Poultry (consumption frequency)** | **-0.074** | **-0.137** | **-0.011** | **0.021** | * |
| **Score of consumption frequency** | **-0.065** | **-0.128** | **-0.002** | **0.043** | * |
| **Score of residency location** | **0.398** | **0.335** | **0.462** | **≤0.001** | *** |

Legend: *β* – standardized regression coefficient, CI – confidence interval, *p* – significance level, ΣPC1 – molar sum of PCB congeners represented by principal component 1 (PCB 138, PCB 153, PCB 156, PCB 157, PCB 167, PCB 170, PCB 180, PCB 187+182, PCB 189, PCB 194, PCB 196+203, and PCB 199+201), * p≤0.05, ** p≤0.01, *** p≤0.001

**Table S2** Stepwise regression model of predicting variables for molar sum of PCB congeners represented by PC2.

| **Model: ΣPC2** | ***β*** | **95% CI** | | ***p*** |  |
| --- | --- | --- | --- | --- | --- |
| **Age** | **0.094** | **0.024** | **0.163** | **0.008** | ** |
| Active smoking | -0.068 | -0.138 | 0.002 | 0.057 |  |
| **Second-hand smoking** | **-0.084** | **-0.154** | **-0.015** | **0.017** | * |
| **Alcohol consumption** | **0.074** | **0.004** | **0.144** | **0.039** | * |
| **Working in industry** | **0.146** | **0.076** | **0.217** | **≤0.001** | *** |
| **Bacon (consumption frequency)** | **-0.070** | **-0.139** | **-0.002** | **0.045** | * |
| Fish (origin) | -0.057 | -0.126 | 0.012 | 0.103 |  |
| **Poultry (origin)** | **-0.097** | **-0.167** | **-0.028** | **0.006** | ** |
| **Score of residency location** | **0.448** | **0.377** | **0.518** | **≤0.001** | *** |

Legend: *β* – standardized regression coefficient, CI – confidence interval, *p* – significance level, ΣPC1 – molar sum of PCB congeners represented by principal component 2 (PCB 28, PCB 52, and PCB 101), * p≤0.05, ** p≤0.01, *** p≤0.001

**Table S3** Stepwise regression model of predicting variables for molar sum of PCB congeners represented by PC3.

| **Model: ΣPC3** | ***β*** | **95% CI** | | ***p*** |  |
| --- | --- | --- | --- | --- | --- |
| **Age** | **0.418** | **0.356** | **0.481** | **≤0.001** | *** |
| Second-hand smoking | -0.050 | -0.113 | 0.012 | 0.116 |  |
| **Alcohol consumption** | **0.091** | **0.028** | **0.153** | **0.005** | ****** |
| Fish (origin) | -0.052 | -0.114 | 0.011 | 0.104 |  |
| Score of consumption frequency | -0.050 | -0.113 | 0.013 | 0.121 |  |
| Score of food origin | -0.052 | -0.115 | 0.012 | 0.109 |  |
| **Score of residency location** | **0.436** | **0.373** | **0.500** | **≤0.001** | *** |

Legend: *β* – standardized regression coefficient, CI – confidence interval, *p* – significance level, ΣPC1 – molar sum of PCB congeners represented by principal component 3 (PCB 74, PCB 99, PCB 105, PCB 114, and PCB 118), ** p≤0.01, *** p≤0.001
